# Association Between Oral Microbiota and Human Brain Glioma Grade: A Case-Control Study

**DOI:** 10.3389/fmicb.2021.746568

**Published:** 2021-10-18

**Authors:** Yuqi Wen, Le Feng, Haorun Wang, Hu Zhou, Qianqian Li, Wenyan Zhang, Ming Wang, Yeming Li, Xingzhao Luan, Zengliang Jiang, Ligang Chen, Jie Zhou

**Affiliations:** ^1^Department of Neurosurgery, The Affiliated Hospital of Southwest Medical University, Luzhou, China; ^2^Sichuan Clinical Medical Research Center for Neurosurgery, Luzhou, China; ^3^Department of Prosthodontics, The Affiliated Stomatology Hospital of Southwest Medical University, Luzhou, China; ^4^School of Life Sciences, Westlake University, Hangzhou, China; ^5^Key Laboratory of Growth Regulation and Translational Research of Zhejiang Province, School of Life Sciences, Westlake University, Hangzhou, China; ^6^Neurological Diseases and Brain Function Laboratory, The Affiliated Hospital of Southwest Medical University, Luzhou, China; ^7^Academician (Expert) Workstation of Sichuan Province, The Affiliated Hospital of Southwest Medical University, Luzhou, China

**Keywords:** glioma, malignant grade, oral microbiota, isocitrate dehydrogenase 1 mutation, human cohort

## Abstract

Gliomas are the most prevalent form of primary malignant brain tumor, which currently have no effective treatments. Evidence from human studies has indicated that oral microbiota is closely related to cancers; however, whether oral microbiota plays a role in glioma malignancy remains unclear. The present study aimed to investigate the association between oral microbiota and grade of glioma and examine the relationship between malignancy-related oral microbial features and the isocitrate dehydrogenase 1 (IDH1) mutation in glioma. High-grade glioma (HGG; *n*=23) patients, low-grade glioma (LGG; *n*=12) patients, and healthy control (HCs; *n*=24) participants were recruited for this case-control study. Saliva samples were collected and analyzed for 16S ribosomal RNA (rRNA) sequencing. We found that the shift in oral microbiota β-diversity was associated with high-grade glioma (*p*=0.01). The phylum Patescibacteria was inversely associated with glioma grade (LGG and HC: *p*=0.035; HGG and HC: *p*<0.01). The genera *Capnocytophaga* (LGG and HC: *p*=0.043; HGG and HC: *p*<0.01) and *Leptotrichia* (LGG and HC: *p*=0.044; HGG and HC: *p*<0.01) were inversely associated with glioma grades. The genera *Bergeyella* and *Capnocytophaga* were significantly more positively correlated with the IDH1 mutation in gliomas when compared with the IDH1-wild-type group. We further identified five oral microbial features (*Capnocytophaga Porphyromonas*, *Haemophilus*, *Leptotrichia*, and *TM7x*) that accurately discriminated HGG from LGG (area under the curve [AUC]: 0.63, 95% confidence interval [CI]: 0.44–0.83) and HCs (AUC: 0.79, 95% CI: 0.68–0.92). The functional prediction analysis of oral bacterial communities showed that genes involved in cell adhesion molecules (*p*<0.001), extracellular matrix molecule-receptor interaction (*p*<0.001), focal adhesion (*p*<0.001), and regulation of actin cytoskeleton (*p*<0.001) were associated with glioma grades, and some microbial gene functions involving lipid metabolism and the adenosine 5'-monophosphate-activated protein kinase signaling pathway were significantly more enriched in IDH1 mutant gliomas than compared with the IDH1-wild-type gliomas. In conclusion, our work revealed oral microbiota features and gene functions that were associated with glioma malignancy and the IDH1 mutation in glioma.

## Introduction

Gliomas, the most common primary tumor of the central nervous system, are stratified into grades 1–4 based on the histological features defined by the World Health Organization (WHO; Louis et al., 2016). This classification system has been transformed into a molecular feature-based classification system and can be used for the formulation of targeted therapeutic methods, which have been shown to have a higher level of prognostic accuracy (Louis et al., 2016, 2020; Brat et al., 2020). WHO grades 1–2 gliomas (low-grade gliomas; LGG) exhibit low aggressive tendencies and have a better prognosis, whereas WHO grades 3–4 gliomas (high-grade gliomas; HGG) have a high rate of deterioration and a poor prognosis (Louis et al., 2016). Evidence from human studies has indicated that oral microbiota is closely related to cancers (Michaud et al., 2013; Fan et al., 2018); however, whether oral microbiota plays a role in glioma malignancy remains unclear.

The presence of mutant forms of isocitrate dehydrogenase 1 (IDH1) is a key factor in determining the prognosis of patients with gliomas. Generally speaking, glioma patients with the IDH1 mutation have a more favorable prognosis, and the mutation is frequently expressed in patients with an LGG but rarely detected in patients with a WHO grade 4 glioma (Ceccarelli et al., 2016). Oral microbial-produced substances may be carcinogenic (Kurkivuori et al., 2007; Meurman and Uittamo, 2008). Therefore, the mechanism underlying the link between the IDH1 mutation and better prognosis may involve the variation in the composition and function of oral microbiota. Currently, the relationship between oral microbiota and the glioma IDH1 mutation is uncertain.

Therefore, we aimed to investigate the association between oral microbiota and glioma grade and examine the relationship between the composition and functional features of malignancy-related oral microbiota and the glioma IDH1 mutation.

## Materials and Methods

### Study Subjects and Study Design

The cohort was a prospective cohort that included 59 participants of Han Chinese ethnicity. This study was a cross-sectional analysis of the retrospective cohort at baseline. Briefly, 59 participants aged 20–74years who were living in Luzhou City, Southern China, were recruited by the Department of Neurosurgery, Affiliated Hospital of Southwest Medical University, between April 2019 and October 2020. We collected sociodemographic, lifestyle, and dietary factor information. Anthropometric parameters, which included weight and height, were measured by trained nurses. The glioma IDH1 mutation was assessed by postoperative pathological diagnosis. Clinical physiological variables (blood glucose value) were measured using a blood glucose monitor. Oral saliva samples of participants were collected during participants’ visits to the study site. We excluded participants who had been physiologically diagnosed with a non-glioma and had not received surgery. Finally, 59 participants were included in the present analysis (see inclusion and exclusion criteria for further details; Supplementary Methods; Supplementary Figure 1).

Glioma grades were assessed according to the 2016 WHO Classification of Tumors of the Central Nervous System. Participants were divided into three groups: (i) healthy controls (HCs; *n*=24), (ii) LGG group (*n*=12), and (iii) HGG group (*n*=23) according to the criteria for glioma grades. HCs were recruited from patients’ families, such as their spouses and parents. The study protocol was approved by the ethics committee of the Affiliated Hospital of Southwest Medical University (No. KY2019030), and all participants provided written informed consent.

### Sample Collection and DNA Extraction

Participants were asked to refrain from drinking, eating, brushing teeth, or smoking on the morning of the study visit and to rinse out impurities in the mouth with sterile saline. During the visit to the study center, participants were provided with a saliva sampler and detailed instructions for the saliva sample collection. Briefly, each participant collected their saliva sample by natural secretion, which was kept in the mouth for 3min. They then spat 20ml of saliva into a 50-ml sterile centrifugal tube. All saliva samples were immediately frozen and stored in a −80°C freezer.

All DNA extraction steps were performed in a biosafety cabinet. Oral saliva DNA was isolated using a QIAamp Fast DNA Stool Mini Kit (QIAGEN, Hilden, Germany) according to standard protocols. NanoDrop was used to quantitatively detect the concentration of DNA in each sample, and 1% agarose gel electrophoresis was used to evaluate the integrity of DNA. The DNA samples that met the quality requirements (i.e., A260/280=1.8–2.8, total DNA>500ng, the main strip of the gel was complete without an obvious tail) were frozen at −80°C for subsequent analyses, and non-conforming samples were discarded.

### Oral Microbiota Profiling Using 16S rRNA Sequencing

The 16S ribosomal RNA (rRNA) gene amplification procedure was divided into two polymerase chain reaction (PCR) steps. For the first PCR reaction, the V3-V4 hypervariable region of the 16S rRNA gene was amplified from the genomic DNA using primers 338F(ACTCCTACGGGAGGCAGCAG) and 806R(GGACTACHVGGGTWTCTAAT). The amplification products were purified by gel extraction (AxyPrep DNA Gel Extraction Kit, Axygen Biosciences, Union City, CA, United States) according to manufacturer instructions. The concentration of the pooled libraries was determined using the Qubit quantification system. Amplicon sequencing was performed on the MiSeq PE250 platform (Illumina, San Diego, California, United States). Automated cluster generation and 2×250bp paired-end sequencing with dual-index reads were performed.

### 16S rRNA Gene Sequencing Bioinformatics Analysis

Sequence analysis was performed using the Quantitative Insights into Microbial Ecology Pipeline (QIIME) software version 2-2020.2 (Bolyen et al., 2019). The divisive amplicon denoising algorithm (DADA2; Callahan et al., 2016) was used for amplicon sequence variant (ASV) clustering equaling 100%. A representative sequence was selected for each ASV, and the SILVA reference database was used to annotate taxonomic information. The absolute abundance table was extracted from the pipeline and converted into relative abundances by normalization for analyzing the composition of gut microbiota by QIIME2 for downstream analysis.

### Statistical Analyses

For comparisons between the three groups, we used the chi-square test for categorical variables and analysis of variance (ANOVA) for continuous variables. We examined the associations between glioma grade and oral microbial *α*-diversity indices (Shannon, ACE, and Chao1 indices and Good’s coverage), which were estimated based on species richness in the ASV subsample table. The association between glioma grade and β-diversity (between-subject diversity) dissimilarity, based on ASV-level Bray-Curtis distance, was analyzed using permutational ANOVA (999 permutations), adjusted for age, sex, and body mass index (BMI).

We used Wilcoxon rank-sum tests to determine oral microbiota and microbial function associations with glioma grade (a *value of p*≤0.05 was considered statistically significant). The Benjamini-Hochberg method was used to control for false discovery rate (FDR). Receiver operator characteristic curves based on the identified oral microbial features were used to discriminate different glioma grade patients from HCs. The true positive rate (sensitivity) was plotted against the false positive rate (100%−specificity), and the area under the curve (AUC) values were reported with 95% confidence intervals (CI) as an estimate of diagnostic utility.

We examined the associations between the composition and functional features of malignancy-related oral microbiota and the glioma IDH1 mutation using multivariable linear regression, adjusted for age, sex, and BMI. The Benjamini-Hochberg method was used to control for FDR. We also conducted stratified analyses for smoking and alcohol status. Analyses were carried out using R statistical software (version 3.3.1, R Foundation). A *value of p* <0.05 was considered statistically significant.

## Results

### Characteristics of Study Participants

The demographic characteristics of participants are shown in Table 1. The mean (standard deviation) age was 46.12 (12.69) years, and 47.46% were women (Table 1).

**Table 1:** Clinical characteristics of the study population in this study.

Characteristics		HCs (*N*=24)	Glioma grade	
			LGG (*N*=12)	HGG (*N*=23)
Age (years)				
Range		32–57	20–61	21–74
Mean±SD		45.29±6.72	37.67±13.49	51.39±14.75
Gender				
Male		10 (41.67%)	8 (66.67%)	13 (56.52%)
Female		14 (58.33%)	4 (33.33%)	10 (43.48%)
BMI (kg/m^2^)				
Range		19.59–37.78	18.51–25.77	18.07–28.69
Mean±SD		25.59±3.42	22.18±1.90	22.99±2.90
Blood glucose				
Range		—	4.4–14.9	4.77–22.5
Mean±SD		—	7.0±3.06	8.13±3.63
Excrement regularity				
Yes		17 (70.83%)	9 (75%)	16 (69.57%)
No		7 (29.17%)	3 (25%)	7 (30.43%)
Brushing habits				
Numbers	0 time	0 (0%)	3 (25%)	3 (13.05%)
		1 time	20 (83.33%)	7 (58.33%)	13 (56.52%)
		2 times	4 (16.67%)	2 (16.67%)	7 (30.43%)
Time		<2min	21 (87.5%)	6 (50%)	22 (95.65%)
		> 2min	3 (12.5%)	6 (50%)	1 (4.35%)
Tooth missing				
Yes		7 (29.17%)	5 (41.67%)	9 (39.13%)
No		17 (70.83%)	7 (58.33%)	14 (60.87%)

In this study, according to the recruitment standard, 59 cases including 24 HCs and 35 glioma patients were included. Glioma patients were divided into LGG (*N*=12), HGG (*N*=23). HCs, healthy controls; LGG, low-grade glioma; HGG, high-grade glioma.

### Association Between Glioma Grade and Oral Microbiota Diversity

**Figure 1:**
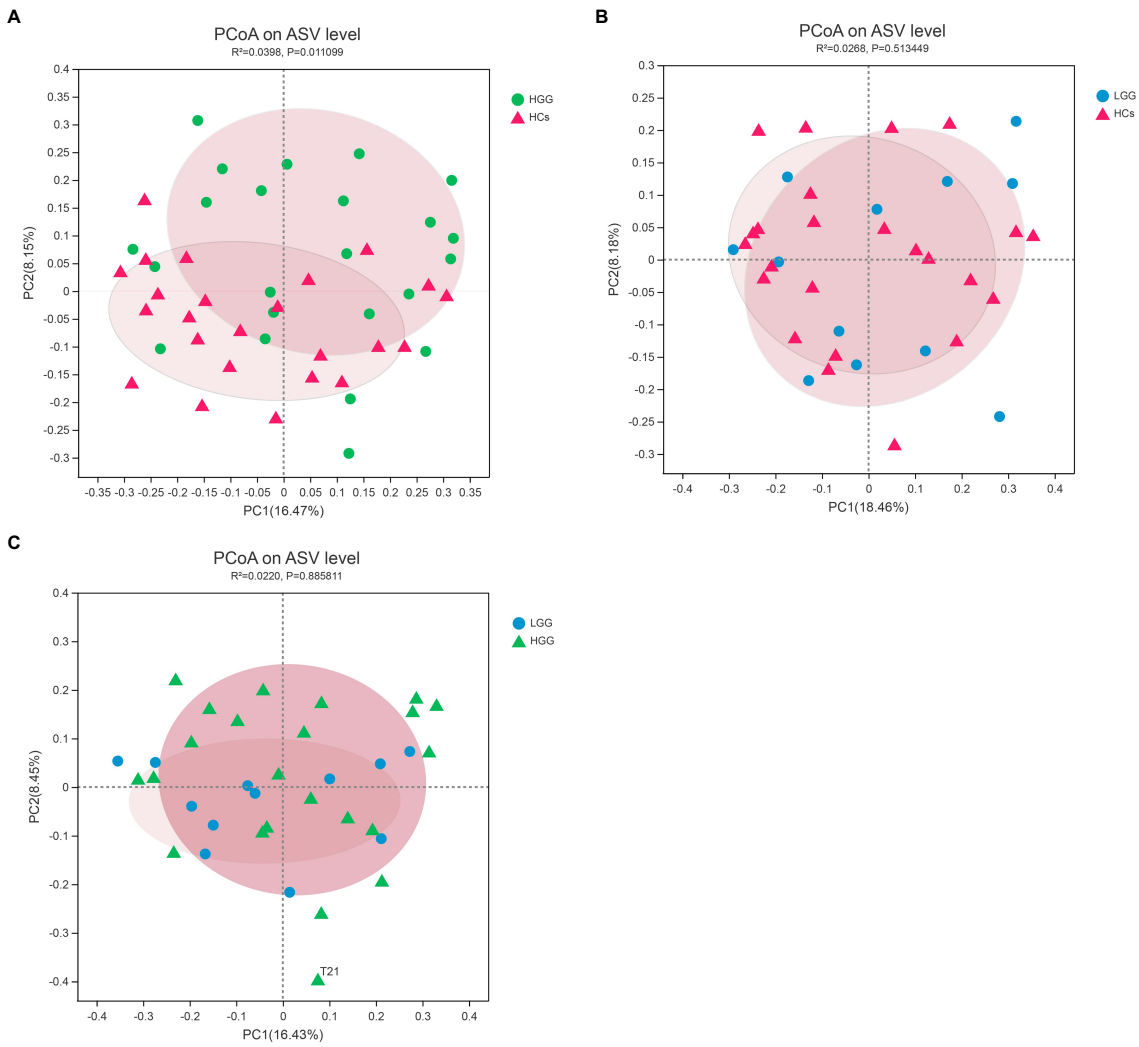
Principal component analysis (PCoA) plots based on the Bray-Curtis distances showed distinct clusters in **(A)** HCs&HGG, **(B)** HCs&LGG, and **(C)** HGG&LGG. The individual samples are color-coded to indicate HCs (red), LGG (blue), and HGG (green). HCs, healthy controls; HGG, high-grade glioma; LGG, low-grade glioma.

We first investigated the associations between glioma grade and microbiome α-/β-diversity. A significant difference in microbial β-diversity (*p*=0.01) was found between the HGG and HC groups (Figure 1A), whereas no significant difference was found between the LGG and HC groups (*p*=0.51; Figure 1B), and no significant difference was found between the LGG and HGG groups (*p*=0.89; Figure 1C). There were no significant differences in measures of α-diversity between the glioma groups and the HCs [HGG and HC: Shannon index: *p*=0.34, phylogenetic diversity (PD): *p*=0.86; LGG and HC: Shannon index: *p*=0.91, PD: *p*=0.21].

### Glioma Grades Were Associated With Oral Microbiota Composition and Gene Function

**Figure 2:**
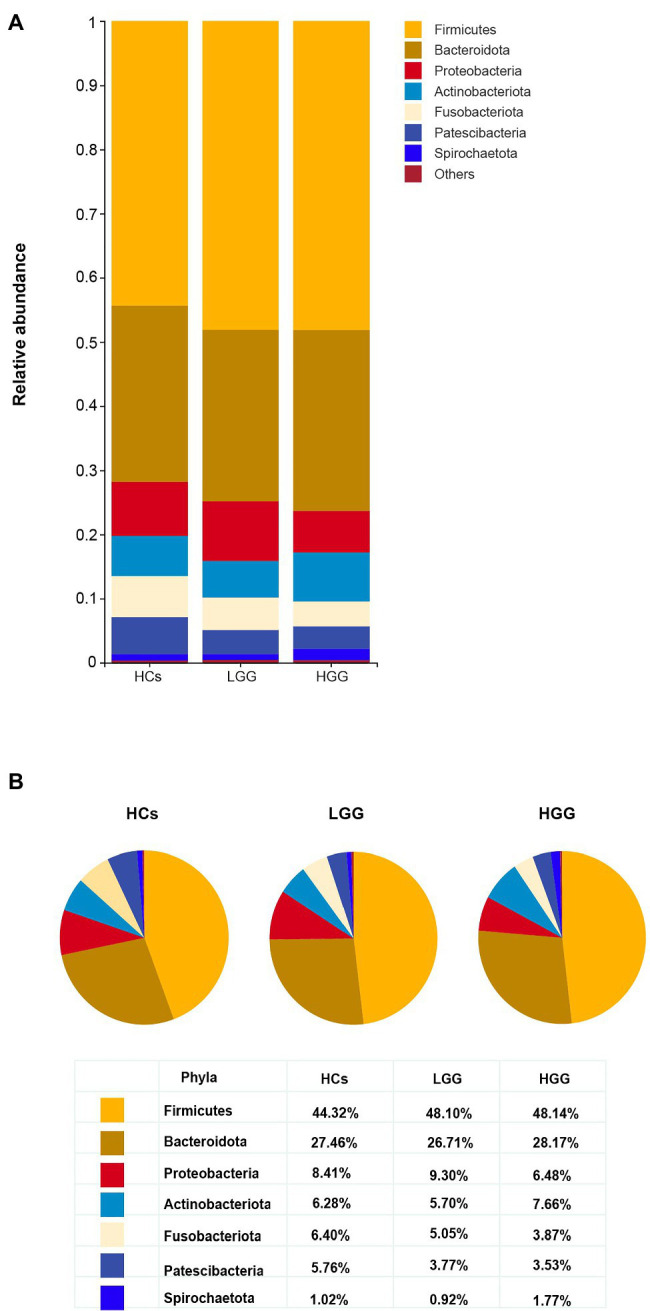
Comparison of the structures of the oral microbiome at the phylum level among HCs, LGG, and HGG. **(A)** The seven most abundant bacteria phylum in the oral microbiome, “Others” represents the bacteria with the relative abundance of less than 1%; **(B)** the proportion of Firmicutes, Bacteroidetes, Proteobacteria, Fusobacteria, Actinobacteria, Patescibacteria, and Spirochaetota in each group at the phylum level.

**Figure 3:**
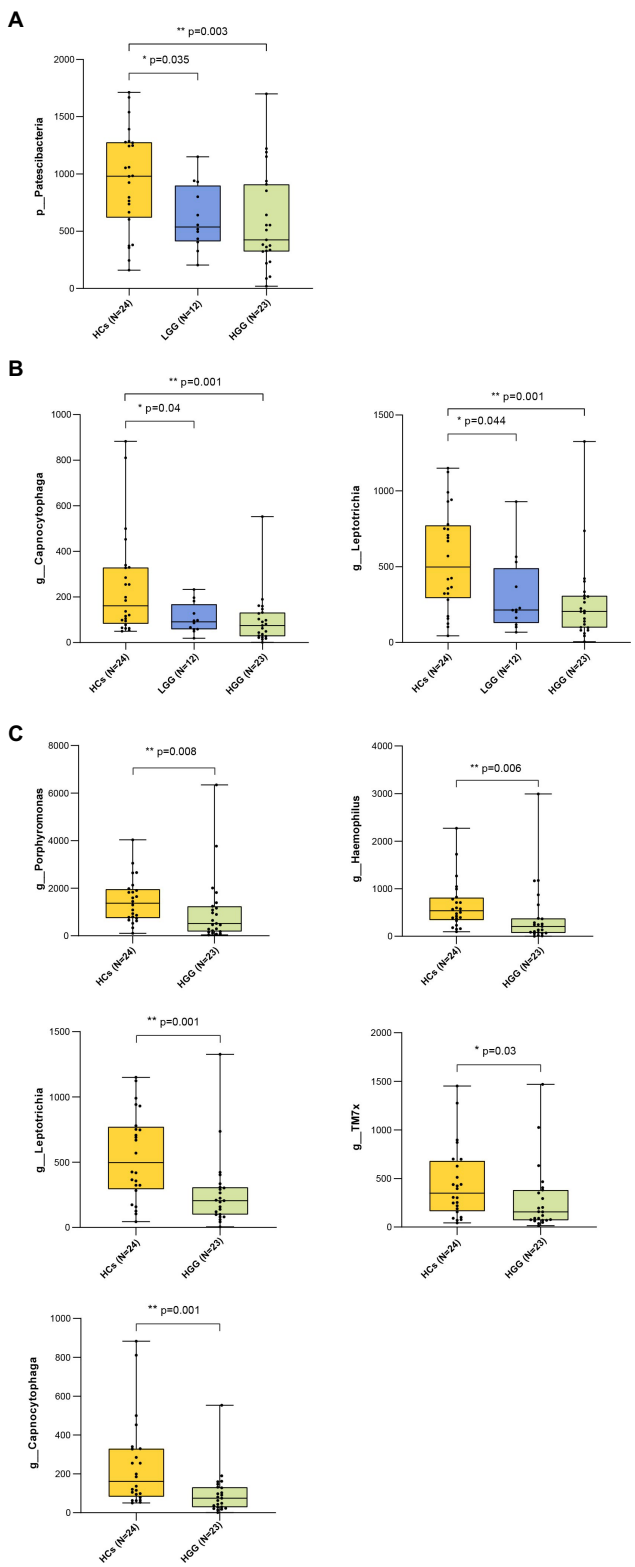
Bacteria taxonomic profiling of oral microbiome from healthy controls and glioma patients. **(A)** Relative abundance of the pylum Patescibacteria in HCs, LGG, and HGG; **(B)** relative abundance of *genera* Capnocytophaga and Leptotrichia in HCs, LGG, and HGG; **FIGURE 3(C)** relative abundance of genera Porphyromonas, Haemophilus, Leptotrichia, TM7x, Capnocytophaga in HCs, and HGG. The box plot represented the relative abundance of bacteria genus in HCS, LGG, and HGG. The *p* value was calculated by non-parametric Mann-Whitney U test. Each box plot represents the median, interquartile range, minimum, and maximum values. *p* value <0.05 indicated the statistical significance. HCs, healthy controls; LGG, low-grade glioma; HGG, high-grade glioma.

Our results showed that 99.57% of the oral microbiota was aligned to seven phyla, which included *Firmicutes*, *Bacteroidetes*, *Proteobacteria*, *Actinobacteria*, *Fusobacteria*, *Patescibacteria*, and *Spirochaetota* (Figures 2A,B). The abundance of *Patescibacteria* decreased significantly with increasing malignancy of glioma from LGG (*p*=0.035) to HGG (*p*<0.01), compared with HCs (Figure 3A). The proportions of the other six phyla (*Firmicutes*, *Bacteroidetes*, *Proteobacteria*, *Actinobacteria*, *Fusobacteria*, and *Spirochaetota*) were not associated with glioma grade (Supplementary Figure 2).

**Figure 4:**
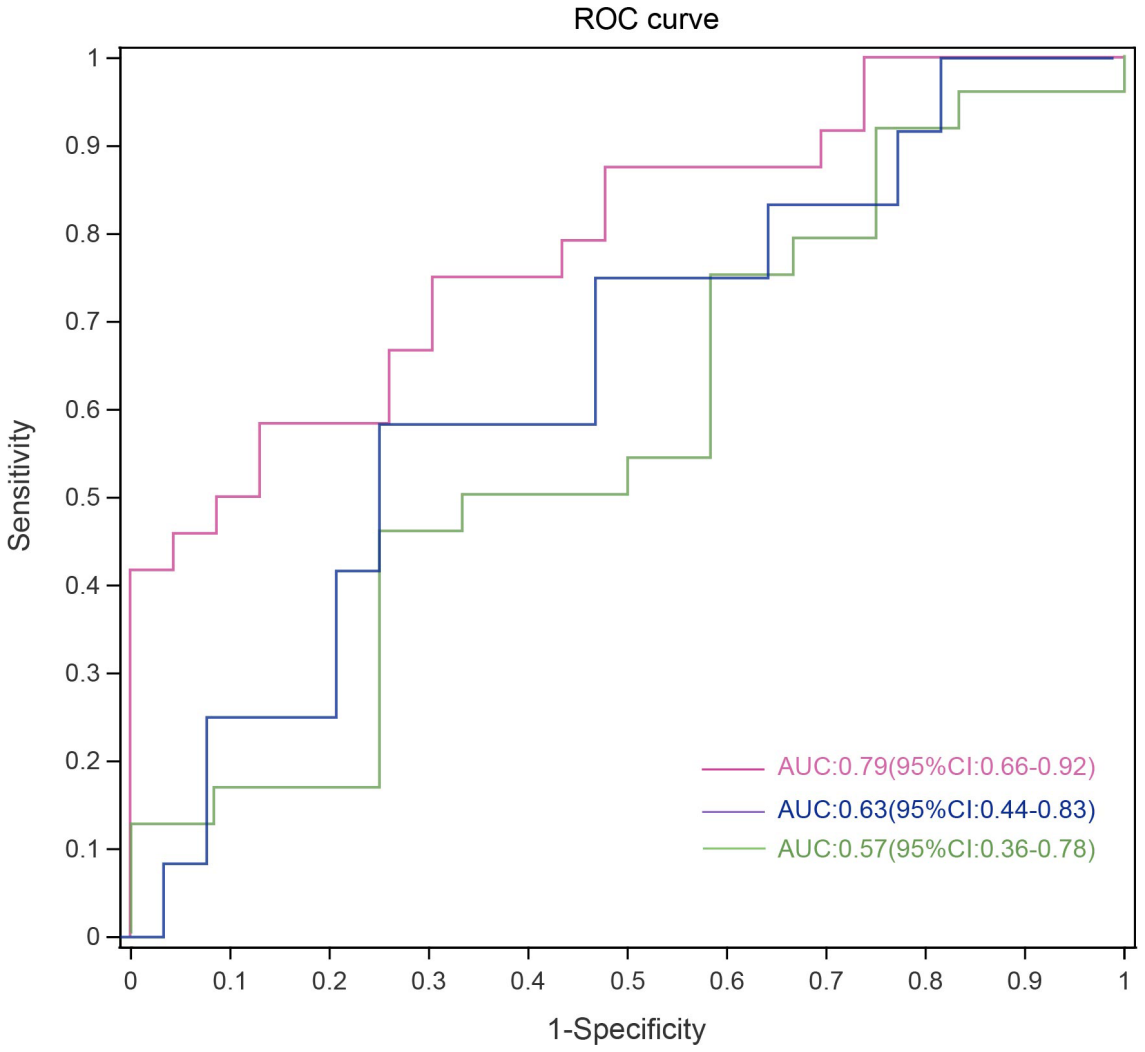
Disease malignancy based on oral microbiome signature. ROC curves evaluating ability to predict tumor malignancy patients based on 5 significantly altered genera. Each curve represents the sensitivity and specificity to distinguish subjects with HGG (red line), LGG (green line) from HCs, while the blue line represents sensitivity and specificity between LGG and HGG. ROC, receiver operating characteristic; HGG, high-grade glioma; LGG, low-grade glioma; HCs, healthy controls.

The ASVs were assigned to 181 individual genera of which 18 were present in all samples with a relative abundance of more than 1% in at least one sample (Supplementary Figures 3A,B). The genera *Capnocytophaga* (LGG and HC: *p*=0.043; HGG and HC: *p*<0.01) and *Leptotrichia* (LGG and HC: *p*=0.044; HGG and HC: *p*<0.01) were inversely associated with glioma grade (Figure 3B). Five oral microbial features [*Porphyromonas* (*p*<0.05), *Haemophilus*, *Leptotrichia* (*p*<0.05), *TM7x* (*p*<0.05), and *Capnocytophaga* (*p*<0.05)] were significantly lower in the HGG group compared with the HC group (Figure 3C). The five oral microbial features (*Porphyromonas*, *Haemophilus*, *Leptotrichia*, *TM7x*, and *Capnocytophaga*) accurately discriminated the HGG group from HCs (AUC: 0.79, 95% CI: 0.68–0.92; Figure 4 and Supplementary Table 1). We also use bacterial marker panels to discriminate the HGG group from the LGG group (AUC: 0.63, 95% CI: 0.44–0.83) and the LGG group from HCs (AUC: 0.57, 95% CI: 0.36–0.78; Figure 4).

**Figure 5:**
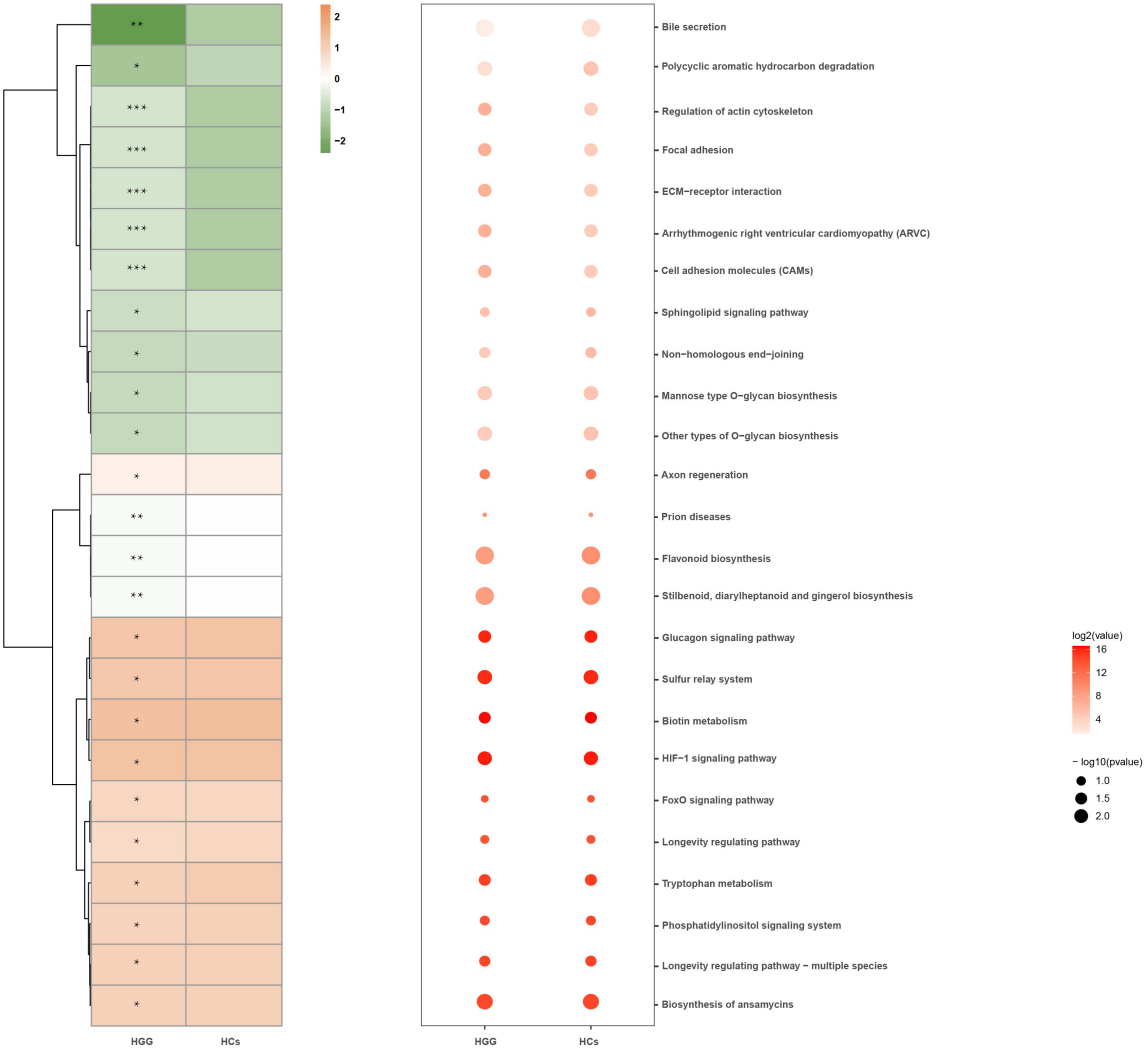
Microbial functions altered in the HCs and HGG. Heat map showing the medial abundance of all significant modules as determined by PICRUSt analysis at HCs and HGG. ^*^ *p*<0.05, ^**^p<0.01, ^***^p<0.001. PICRUSt, Phylogenetic Investigation of Communities by Reconstruction of Unobserved States; HCs, healthy controls; HGG, high-grade glioma.

We used the Phylogenetic Investigation of Communities by Reconstruction of Unobserved States (PICRUSt) to predict the oral microbiome functions *via* the saliva microbiome data sets. Signaling molecules and interactions, such as cell adhesion molecules (CAMs), extracellular matrix (ECM)-receptor interactions, cellular community-eukaryotes (focal adhesion), and actin cytoskeleton regulation, were positively associated with glioma grade, while the polycyclic aromatic hydrocarbon degradation and the Bile secretion were inversely associated with HCs (Figure 5).

### Glioma IDH1 Mutation Was Associated With Malignancy-Related Oral Microbiota and Gene Function

**Figure 6:**
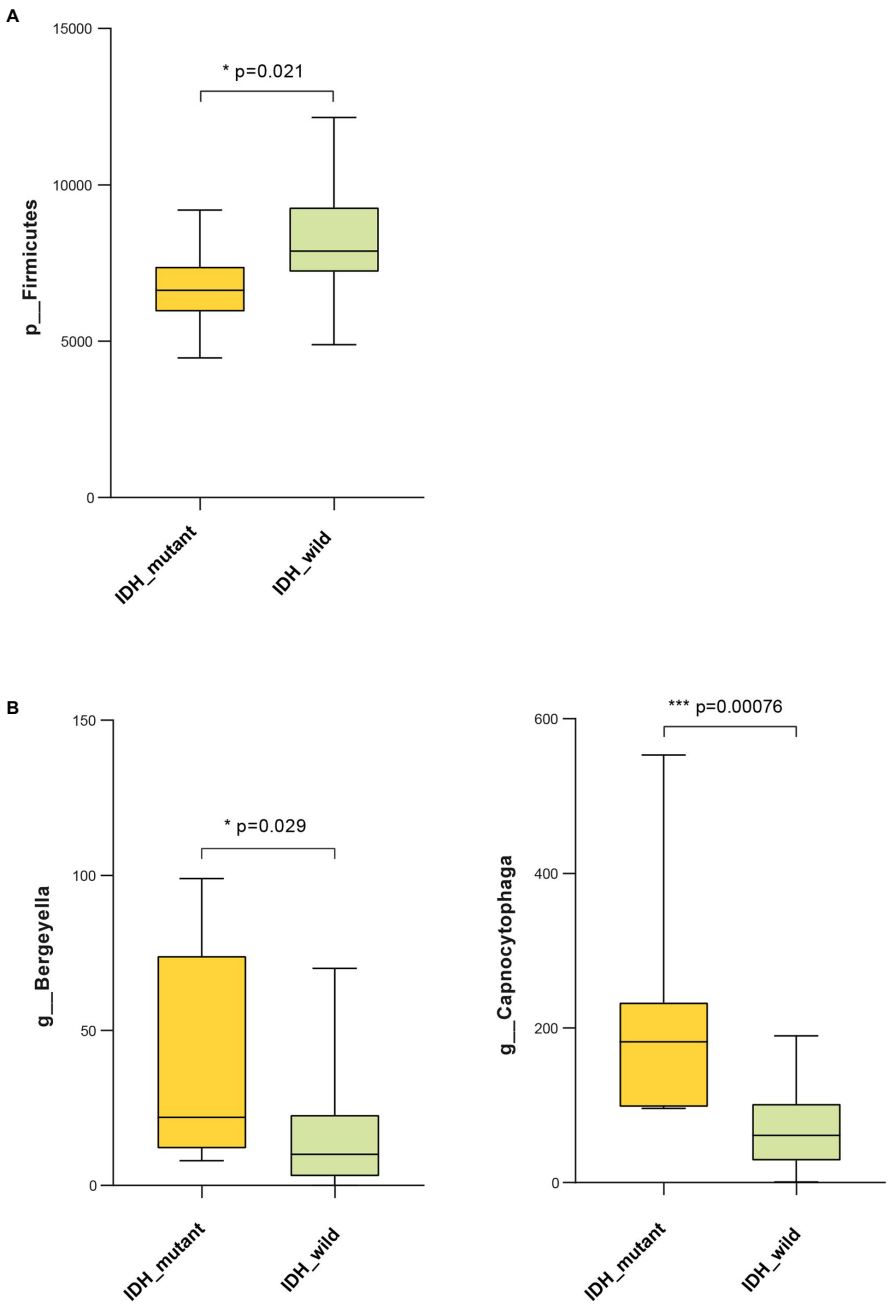
Bacteria taxonomic profiling of oral microbiome from IDH1-mutant group and IDH1-wild group. **(A)** Relative abundance of phylum Proteobacteria in IDH1-mutant group and IDH1-wild group; **(B)** relative abundance of genera Bergeyella and Capnocytophaga in IDH1-mutant group and IDH1-wild group. Cross coordinates represent different group names and longitudinal represent the abundance of a species in different groups. The *p* value was calculated by non-parametric Mann-Whitney U test. Each box plot represents the median, interquartile range, minimum, and maximum values. IDH1, isocitrate dehydrogenase 1.

Results showed that the abundance of *Firmicutes* was significantly lower in the IDH-mutant samples compared with that of the IDH-wild-type samples at the phylum level (Figure 6A). The genus-level profiling showed that the abundance of *Bergeyella* and *Capnocytophaga* was significantly positively correlated with the IDH-mutant samples (Figure 6B). We also used PICRUSt to predict Kyoto Encyclopedia of Genes and Genomes (KEGG)-based functional orthologs between the IDH-mutant and IDH-wild-type groups to characterize the functional alterations, which were inferred from the 16S rRNA gene sequencing data. We found 82 pathways that were significantly greater in the IDH-mutant group compared with those of the IDH-wild-type group using the Mann-Whitney U test. Our result revealed that several metabolic-related pathways, such as lipid metabolism (linoleic acid, ether lipid metabolism, fatty acid biosynthesis, glycerophospholipid, and biosynthesis of unsaturated fatty acids), fatty acid metabolism (alpha-linolenic acid), amino acid metabolism (tyrosine metabolism, tryptophan metabolism, lysine degradation, and glycine, serine, and threonine metabolism), carbohydrate metabolism (inositol phosphate metabolism), and cofactors and vitamin metabolism (retinol metabolism), were significantly higher in the IDH-mutant group compared with those of the IDH-wild-type group. Moreover, signal transduction, such as the adenosine 5′-monophosphate-activated protein kinase (AMPK) signaling pathway, the phosphatidylinositol signaling system, the sphingolipid signaling pathway, and the phospholipase D signaling pathway, was more enriched in the IDH-mutant group than in the IDH-wild-type group (Supplementary Figure 4).

## Discussion

To our knowledge, this is the first comprehensive clinical 16S rRNA sequencing data set to characterize the community features of oral microbiota in different glioma grades. We found that the shift in oral microbiota β-diversity was associated with HGG. The phylum *Patescibacteria* was inversely associated with glioma grade, and the genera *Capnocytophaga* and *Leptotrichia* were inversely associated with glioma grade. We identified five oral microbial features (*Porphyromonas*, *Haemophilus*, *Leptotrichia*, *TM7x*, and *Capnocytophaga*) that accurately discriminated patients with HGG from those with LGG and HCs. The gene function of oral bacterial communities was associated with glioma grade. Moreover, the abundance of the phylum *Firmicutes* was significantly negatively correlated with IDH-mutant samples, whereas the genera *Bergeyella* and *Capnocytophaga* were significantly positively correlated with IDH-mutant samples. Several microbial (lipid metabolism, amino acid metabolism, and energy metabolism) and signal transduction (AMPK signaling pathway) pathways were significantly higher in the glioma IDH-mutant group than those in the glioma IDH-wild-type group. Our findings revealed that oral microbiota features and gene functions are associated with glioma malignancy and the IDH1 mutation.

Previous studies have shown that oral microbiome significantly affected the composition of gut microbiome (Hou et al., 2021; Pandya et al., 2021; Park et al., 2021). The oral microbiome and gut microbiome can spread to the brain through cranial nerves or cellular infections or produced certain metabolites to affect the brain by both direct and indirect means (Dominy et al., 2019; Jing et al., 2021; Narengaowa et al., 2021; Vyhnalova et al., 2021; Wang et al., 2021). Our results showed significant differences in oral microflora between HGG patients and HCs; oral *Patescibacteria* was significantly decreasing during the progression of glioma malignancy. Few studies have reported a correlation between oral *Patescibacteria* and human disease. This is the first study examining *Patescibacteria* in oral saliva samples of glioma patients and suggests *Patescibacteria* as a negatively associated risk factor for disease progression from LGG to HGG. Our results highlight the potential of *Patescibacteria* detection as a diagnostic and prognostic determinant for glioma malignancy, but further experimental research to establish the mechanistic basis of these relationships is needed.

In addition, the phylum *Fusobacteriota* was significantly lower in the HGG group than in the HCs. Furthermore, the family *Leptotrichiaceae* (Supplementary Figure 3C) and the genus *Leptotrichia*, which belongs to *Fusobacteriota*, were inversely associated with glioma malignancy. This is consistent with the results of two large, nested, case-control studies in which a greater abundance of *Leptotrichia* was associated with a decreased risk of pancreatic cancer (Michaud et al., 2013; Fan et al., 2018). *Leptotrichia* is considered an opportunistic pathogen and can stimulate human immune system responses (Eribe and Olsen, 2017). Moreover, *Leptotrichia* may elicit the immune response and thus protect against pancreatic carcinogenesis (Inman et al., 2014).

Genus-level analysis showed that a bacterial marker panel with *Capnocytophaga*, *TM7x*, *Porphyromonas*, *Haemophilus*, and *Leptotrichia* had an AUC of 0.79 for discriminating between HGG and HCs. We found that the relative abundance of genus *Capnocytophaga* was inversely associated with glioma malignancy. *Capnocytophaga* is a genus of Gram-negative anaerobes that inhabit the oral cavity (Lopez et al., 2010), which has been reported to be inversely associated with human diseases, such as chorioamnionitis, neonatal infection (Lopez et al., 2010), and lung cancer (Thirumala et al., 2012). Consistent with our results, Hayes et al. reported that a greater abundance of the genus *Capnocytophaga* was significantly associated with a reduced risk of larynx cancer (Hayes et al., 2018). However, the protective mechanism of *Capnocytophaga* for glioma malignancy remains unclear. Previous studies have consistently shown a decrease in *Haemophilus* and *Porphyromonas* in the saliva of patients with cancer compared with that of HCs (Mei et al., 2018; Yang et al., 2018; Lu et al., 2019; Li et al., 2020). In addition, the high abundance of *Haemophilus* and *Porphyromonas* is associated with anticancer-associated immunity (Lu et al., 2019). A high level of antibodies to *Porphyromonas gingivalis* in the serum correlates with a lower risk of pancreatic cancer (Michaud et al., 2013). This study is the first to report the inverse association between the abundance of *TM7x* and glioma malignancy and that *TM7x* is a useful bacteria marker for glioma malignancy diagnosis.

The functional prediction showed that environmental information processing, such as CAMs and ECM-receptor interactions, was significantly higher in HGG patients than in the HCs. Several CAMs, such as neural cell adhesion molecule L1, have been identified to underlie the occurrence of glioma malignancies (Senner et al., 2002; Jiang et al., 2019; Lyu et al., 2021). ECM plays an important role in gliomas, such as in the higher expression of laminin a2 in glioblastoma (Lathia et al., 2012). The key role of the focal adhesion pathway and the level of actin in the cytoskeleton during the migration and invasion of glioblastoma have also been reported (de Semir et al., 2020; Wu et al., 2020).

Our results demonstrated that the genera *Bergeyella* and *Capnocytophaga* were significantly positively correlated with the glioma IDH-mutant, which is consistent with the results of glioma malignancy. The IDH1-mutant plays an important role in glioma cell glucose induction, glutamine metabolism, lipid synthesis, and cell redox regulation (Hartmann et al., 2009; Maus and Peters, 2017; Stuani et al., 2018). Moreover, metabolism deregulation plays an important role in cell growth, proliferation, angiogenesis, and invasion; thus, it has been considered one of the emerging hallmarks of cancer cells (Hanahan and Weinberg, 2011). Recent studies have found that lipid metabolism reprogramming plays a crucial role in the progression of cancer cells, such as in membrane synthesis, energetic production, and signal transduction (Liu et al., 2017). The activation of the AMPK signaling pathway contributes to the anti-inflammatory microenvironment of IDH1-mutated gliomas and thus causes better prognoses in patients with an IDH1-mutated glioma (Han et al., 2019). Our results demonstrated that the malignancy inverse-related microbial gene functions involving lipid metabolism and AMPK signaling pathway were significantly enriched in the IDH-mutant group, suggesting that changes in oral microbial gene functions may underlie the link between the positive association between IDH-mutant gliomas and better prognosis. Moreover, several studies reported that mutant IDH1 in gliomas regulated a number of physiological processes such as inflammatory pathways, metabolic metabolism, hypoxia sensing, histone demethylation, and changes in DNA methylation causing DNA strand breaks, apoptosis, autophagy, and tumor cell death (Gilbert et al., 2014; Viswanath et al., 2018; Zhang et al., 2019; Kadiyala et al., 2021; Pirozzi and Yan, 2021). Therefore, the possible mechanism underlying the association between oral microbiome and IDH1 mutation is that IDH1 mutation specifically selects some oral microbiota, which can produce specific metabolites involved in lipid metabolism and AMPK signaling pathway to regulate intracellular energy homeostasis, increase brain glioma cell apoptosis and autophagy, prevent brain glioma cell proliferation, and contribute to the formation of an anti-inflammatory tumor microenvironment in the brain, and further causes better prognoses in patients with an IDH1-mutated glioma. Certainly, animal and cell experiments are further needed to determine the causality of IDH1 mutation on the oral microbiome under glioma status.

The present study has several strengths. First, to the best of our knowledge, this is the first study examining the role of oral microbiota in glioma malignancy. Second, we developed a novel bacterial marker panel to discriminate HGG patients from LGG patients and HCs. Third, our study revealed that the composition and gene function of oral microbiota were significantly associated with the IDH1 mutation in glioma, which can be used to predict the prognosis of glioma patients. The present study also has several limitations. First, the sample size was relatively small. This study was a small-sample, single-center study because of the challenges in recruiting this type of cohort and the strict inclusion criteria. However, this also guaranteed the consistency of the sample. Second, although we demonstrated that oral microbiota was associated with glioma malignancy and the IDH mutation, the underlying causality remains unclear. Finally, no plaque or tongue-coating specimens were included because of the difficulty in collecting such samples.

In summary, the present study indicated that oral microbiota composition and gene functions are significantly associated with glioma malignancy and the IDH1 mutation. We also discovered a microbial biomarker panel to distinguish HGG patients from HCs. Our results suggest that oral microbiota may be an important preventive target to mitigate glioma malignancy and achieve better prognoses for glioma patients.

## Data Availability Statement

The data presented in the study are deposited in the National Library of Medicine repository (https://submit.ncbi.nlm.nih.gov/), BioProject accession number: PRJNA750937 (https://www.ncbi.nlm.nih.gov/bioproject/PRJNA750937).

## Ethics Statement

The studies involving human participants were reviewed and approved by Ethics committee of the Affiliated Hospital of Southwest Medical University (No. KY2019030). The patients/participants provided their written informed consent to participate in this study.

## Author Contributions

YW, LC, and JZ conceived the study and designed the experiments. YW, LF, and HW analyzed and interpreted the data. YW, LF, HW, and ZJ generated the figure, drafted the manuscript, and contributed to critical revision of the manuscript. HZ, QL, WZ, and MW visited patients, collected the data, and critical revision of the manuscript. YL and XL collected the samples and critical revision of the manuscript. YW, LF, and HW revised the manuscript with input from JZ and ZJ. LC and JZ obtained funding and contributed to study supervision. All authors contributed to the article and approved the submitted version.

## Funding

The research was supported by National Natural Science Foundation project, Grant No. 00022986, and technology projects of Sichuan Province and Grant No. 2018JY0403. The research was further supported by Medical Research Fund for Young Scholars of the Sichuan Medical Association, Grant No. Q16076 and Natural Science Foundation of Southwest Medical University, Grant Nos. 2016XNYD217, 2018-ZRQN-032, and 2016LZXNYD-G03.

## Conflict of Interest

The authors declare that the research was conducted in the absence of any commercial or financial relationships that could be construed as a potential conflict of interest.

## Publisher’s Note

All claims expressed in this article are solely those of the authors and do not necessarily represent those of their affiliated organizations, or those of the publisher, the editors and the reviewers. Any product that may be evaluated in this article, or claim that may be made by its manufacturer, is not guaranteed or endorsed by the publisher.

## Supplementary Material

Supplementary Figure 1 Study flow-chart. A total of 59 participants from the cross-sectional study (HCs=24, LGG=12, HGG=24) with 16S oral microbiome profiling were included in the study. *1 patient with LGG was pathologically diagnosed with non-glioma. **1 patient with HGG was not received surgery. Abbreviations: HCs, healthy controls; LG, low glioma group; HGG, high-grade glioma.

Supplementary Figure 2 Bacteria taxonomic profiling at the phylum level of oral microbiome from healthy controls and glioma patients. Box plots show the relative abundance of Firmicutes, Bacteroidetes, Proteobacteria, Actinobacteria, Fusobacteria, and Spirochaetota in HCs, LGG, and HCG. Each box plot represents the median, interquartile range, minimum, and maximum values. The *p* value was calculated by non-parametric Mann-Whitney U test. Value of *p*<0.05 indicated the statistical significance. HCs, healthy controls; HGG: high-grade glioma; LGG: low-grade glioma.

Supplementary Figure 3 Relative abundance of different bacteria genus in healthy and glioma patients. Bacteria taxonomic profiling at the genus level of oral microbiome from healthy controls and glioma patients. **(A)** The 18 bacteria at the genus level in the oral microbiome, “Others” represents the bacteria with the relative abundance of less than 1%. **(B)** The proportion of Streptococcus, Prevotella, Porphyromonas, Veillonella, Haemophilus, Actinomyces, Neisseria, Porphyromonas, and Granulicatella among HGG and HC groups at the genus level; **(C)** the box plots show the relative abundance of Leptotrichiaceae in HCs, LGG, and HGG. Each box plot represents the median, interquartile range, minimum, and maximum values. The *p* value was calculated by non-parametric Mann-Whitney U test. *p* value <0.05 indicated the statistical significance. HCs, healthy controls; LGG: low-grade glioma; HGG: high-grade glioma.

Supplementary Figure 4 Microbial functions altered in the IDH1-mutant group and IDH1-wild-type group. Heat map showing the medial abundance of all significant modules as determined by PICRUSt analysis at IDH1-mutant group and IDH1-wild-type group. Notes: ^*^ *p*<0.05, ^**^ *p*<0.01, ^***^ *p*<0.001. Abbreviations: PICRUSt, Phylogenetic Investigation of Communities by Reconstruction of Unobserved States; HCs, healthy controls; HGG, high-grade glioma. PICRUSt, Phylogenetic Investigation of Communities by Reconstruction of Unobserved States; IDH1, isocitrate dehydrogenase 1; HCs, healthy controls; HGG: high-grade glioma.
