# Supplementary material for: Association Between Oral Microbiota and Human Brain Glioma Grade: A Case-Control Study
Source: Front Microbiol. 2021 Oct 18;12:746568. doi: 10.3389/fmicb.2021.746568 (PMC8558631; doi:10.3389/fmicb.2021.746568)
Supplement: Supplementary file 1 [file Data_Sheet_1.docx]

**Supplementary method.**

Proposed inclusion criteria:

(1) All participants are completed the basic information collection and biological specimen collection.

(2) All participants were not undergoing antibiotics treatment within 2 months before the biological specimens were collected; and known active bacterial, fungal, or viral infections.

(3) All participants had no history of radiotherapy or chemotherapy.

(4) All participants had no history of oral disease or operation.

(5) All participants had no history of central nervous system diseases.

(6) All participants had no history of hypertension, diabetes.

(7) The patients were diagnosed of glioma with head imaging, and ready to receive surgical treatment in our hospital.

Exclusion criteria:

(1) The following diseases were found in this time health check: hypertension. cardiovascular disease; diabetes mellitus.

(2) The patients not received surgical treatment.

Final inclusion criteria:

(1) Participants these meet the proposed inclusion criteria for the proposed group and have passed the exclusion criteria.

(2) The patients have received surgical treatment in our hospital and diagnosed of glioma by postoperative pathological.
