# Supplementary figures and images for: Association Between Oral Microbiota and Human Brain Glioma Grade: A Case-Control Study
Source: Front Microbiol. 2021 Oct 18;12:746568. doi: 10.3389/fmicb.2021.746568 (PMC8558631; doi:10.3389/fmicb.2021.746568)

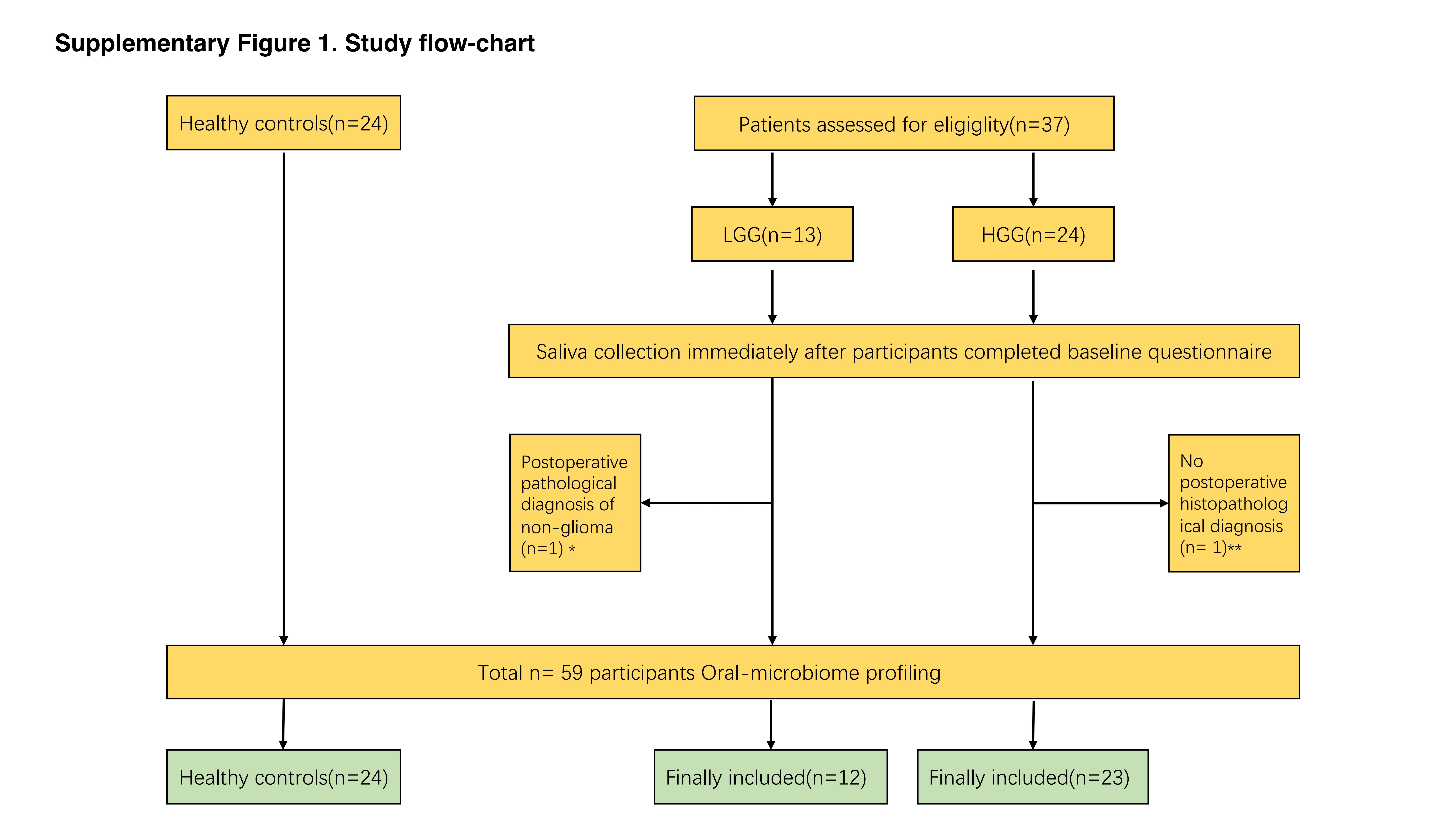

Supplement: Supplementary Figure 1 — Study flow-chart. A total of 59 participants from the cross-sectional study (HCs=24, LGG=12, HGG=24) with 16S oral microbiome profiling were included in the study. *1 patient with LGG was pathologically diagnosed with non-glioma. **1 patient with HGG was not received surgery. Abbreviations: HCs, healthy controls; LG, low glioma group; HGG, high-grade glioma. [file Image_1.JPEG]

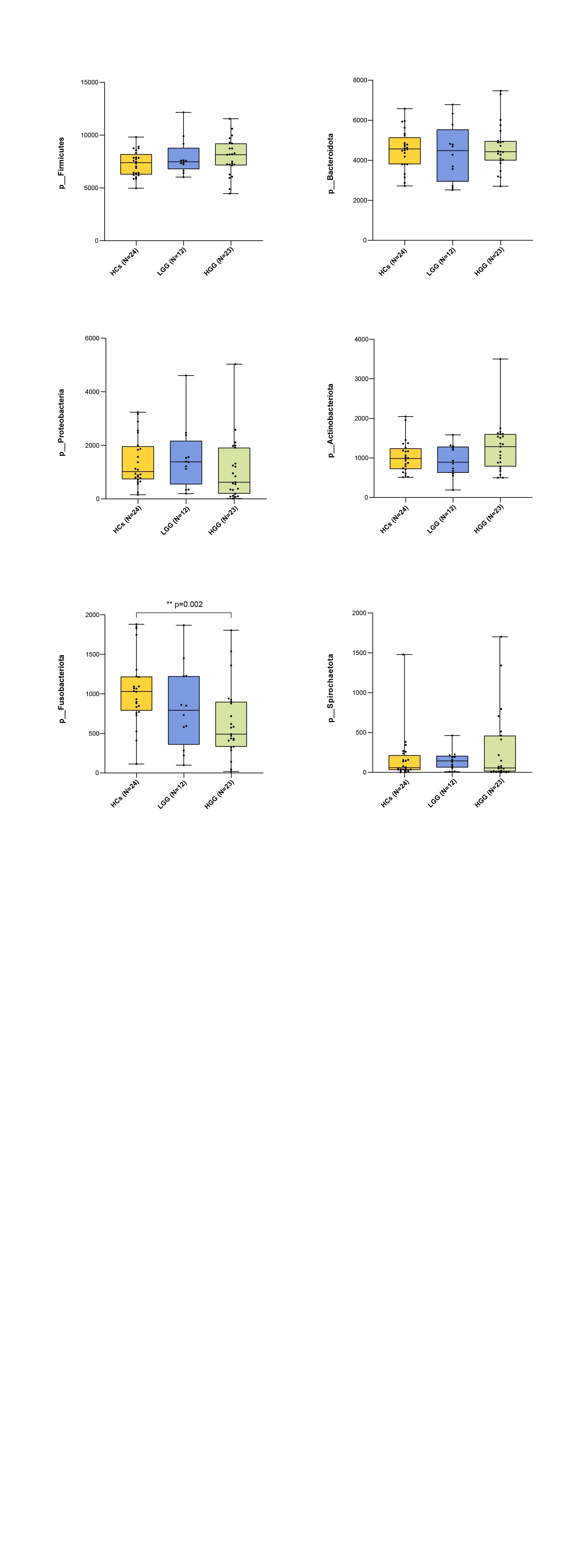

Supplement: Supplementary Figure 2 — Bacteria taxonomic profiling at the phylum level of oral microbiome from healthy controls and glioma patients. Box plots show the relative abundance of Firmicutes, Bacteroidetes, Proteobacteria, Actinobacteria, Fusobacteria, and Spirochaetota in HCs, LGG, and HCG. Each box plot represents the median, interquartile range, minimum, and maximum values. The p value was calculated by non-parametric Mann-Whitney U test. Value of p<0.05 indicated the statistical significance. HCs, healthy controls; HGG: high-grade glioma; LGG: low-grade glioma. [file Image_2.JPEG]

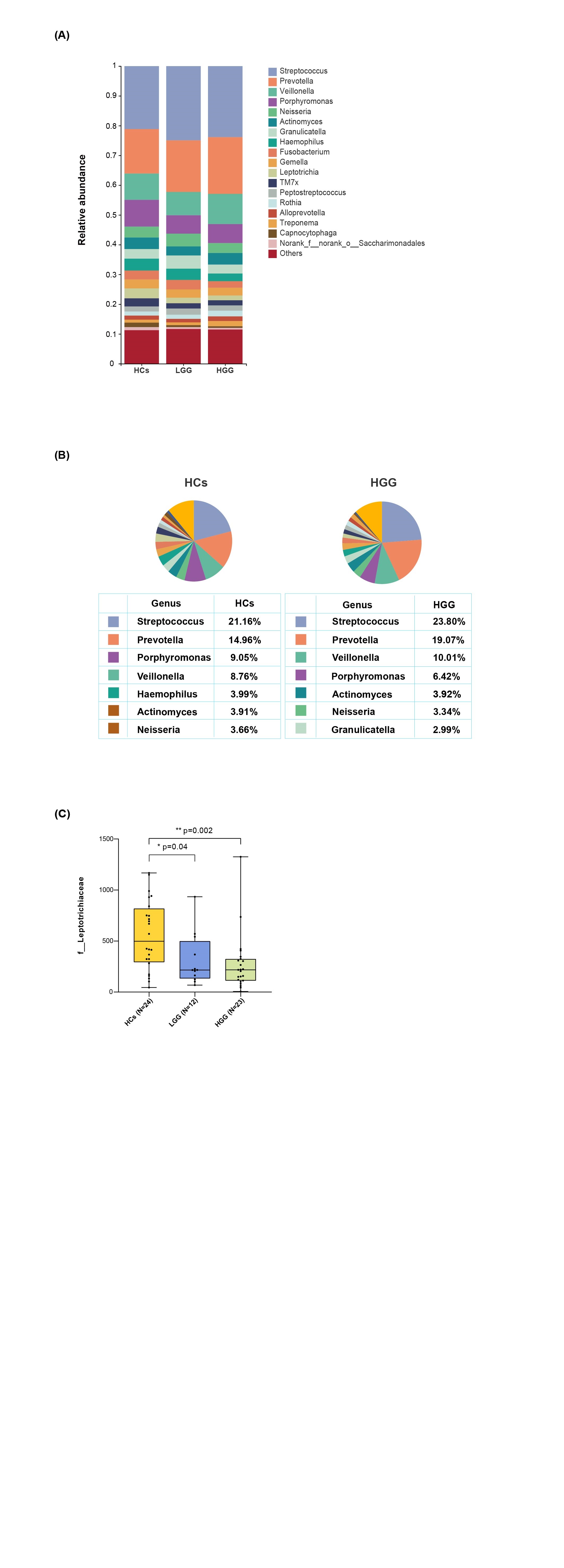

Supplement: Supplementary Figure 3 — Relative abundance of different bacteria genus in healthy and glioma patients. Bacteria taxonomic profiling at the genus level of oral microbiome from healthy controls and glioma patients. (A) The 18 bacteria at the genus level in the oral microbiome, “Others” represents the bacteria with the relative abundance of less than 1%. (B) The proportion of Streptococcus, Prevotella, Porphyromonas, Veillonella, Haemophilus, Actinomyces, Neisseria, Porphyromonas, and Granulicatella among HGG and HC groups at the genus level; (C) the box plots show the relative abundance of Leptotrichiaceae in HCs, LGG, and HGG. Each box plot represents the median, interquartile range, minimum, and maximum values. The p value was calculated by non-parametric Mann-Whitney U test. p value <0.05 indicated the statistical significance. HCs, healthy controls; LGG: low-grade glioma; HGG: high-grade glioma. [file Image_3.JPEG]

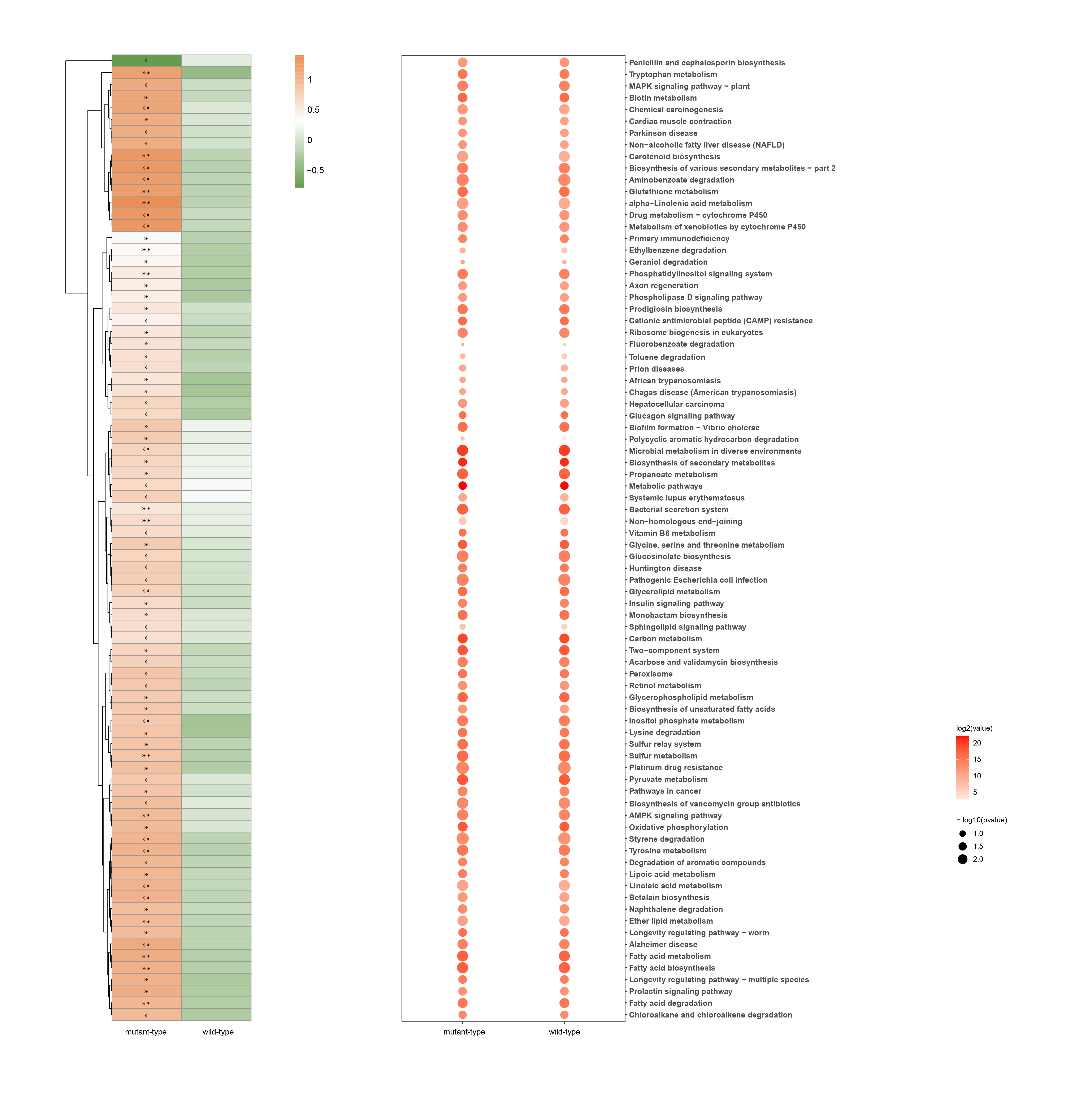

Supplement: Supplementary Figure 4 — Microbial functions altered in the IDH1-mutant group and IDH1-wild-type group. Heat map showing the medial abundance of all significant modules as determined by PICRUSt analysis at IDH1-mutant group and IDH1-wild-type group. Notes: * p<0.05, ** p<0.01, *** p<0.001. Abbreviations: PICRUSt, Phylogenetic Investigation of Communities by Reconstruction of Unobserved States; HCs, healthy controls; HGG, high-grade glioma. PICRUSt, Phylogenetic Investigation of Communities by Reconstruction of Unobserved States; IDH1, isocitrate dehydrogenase 1; HCs, healthy controls; HGG: high-grade glioma. [file Image_4.JPEG]
